# Male fetuses negatively affect the vitality of the litter and the dam’s metabolic and physiological state in multifetal pregnant ewe

**DOI:** 10.1371/journal.pone.0285338

**Published:** 2023-05-09

**Authors:** Tamir Alon, Alexander Rosov, Lila Lifshitz, Uzi Moallem

**Affiliations:** 1 Department of Ruminant Science, Institute of Animal Sciences, The Volcani Center, Rishon LeZion, Israel; 2 Department of Animal Science, the Robert H. Smith Faculty of Agriculture, Food and Environment, The Hebrew University of Jerusalem, Rehovot, Israel; University of Florida, UNITED STATES

## Abstract

In sheep, ~30% of fetuses do not survive till parturition, and 17.7% of the multifetal pregnancies experience partial litter loss (**PLL**). In humans, multifetal pregnancies are associated with a higher risk of perinatal mortality. Therefore, the objectives were to examine the association between partial litter loss, fetal sex, dam’s metabolic and physiological state, and pregnancy outcome in multifetal pregnant ewes. The study includes two parts. The first was a retrospective study, in which we analyzed data of 675 lambings and examined the PLL incidence according to male ratio (**MR**) for all litter sizes (range 2–6). Lambings were categorized as having a low male ratio (**LMR**; <50% males) or a high male ratio (**LMR**; >50% males). In the second part, we monitored 24 ewes from 80 to 138 days in pregnancy every 10 days, and then daily until lambing, by ultrasound scanning for maternal heart rate (**HR**), and Doppler ultrasound for litter vitality. Blood samples were taken from dams on the days of scanning. Male ratio strongly affected PLL, where the general survival rate (for all lambings) was reduced from 90% in LMR lambings to 85% in HMR lambings. The odds ratio for PLL in HMR vs. LMR litters was 1.82. Birth body weight and the survival rate of female was higher in LMR than HMR lambings, with no differences for male lambs in both parameters. In the second part, dams’ HR during the last trimester was 9.4% higher in LMR than in HMR pregnancies, with no differences in fetuses’ HR. The plasma glucose and insulin concentrations were not significantly different between groups, but plasma β-hydroxybutyrate and nonesterified fatty acid concentrations were, respectively, 31% and 20% lower in HMR vs. LMR ewes. In conclusion, male fetuses negatively affect pregnancy outcomes and influence dams’ metabolic and physiological state in sheep.

## Introduction

Generally, in sheep, ~30% of fetuses do not survive until parturition [1]. In parous Romney Marsh ewes, embryonic mortality was estimated in 18, 30, and 140 days in pregnancy (DIP) at 27.0%, 18.1%, and 20.0%, respectively, and for non-parous ewes, it was 20.9%, 15.9%, and 22.0%, respectively [2]. In mixed breeds sheep (mainly Dorset and Suffolk), 19.6% of the fetuses were lost between 25 DIP to birth, and 37% of multifetal pregnancies experienced partial litter loss (PLL), in which 17.7% of them occurred in the last trimester [3]. Multifetal pregnancies were found to be associated with intrauterine growth restriction (IUGR) that affects the lambs’ body weight (**BW**) at birth [4]. It was reported that birth BW for lambs born in liter size of two, three, or four are 0.83, 0.70, and 0.63 of that of singleton lambs, respectively [5]. Fetal growth is regulated by the placental transport of nutrients from the dam through the uteroplacental complex [6]. The number of placentoms per conceptus decreases with the increased number of fetuses; however, although an increase in the placentome size was associated with liter size, it does not achieve the placentome mass of a single conceptus [4]. The decrease in placentome mass may be related to IUGR and low BW at the birth of lambs in multifetal pregnancies.

Twins account for 98% of multifetal pregnancies in humans, associated with a higher risk of perinatal mortality [7]. Single intrauterine fetal demise (**sIUFD**) occurs at a rate of 3.7–6.8% in twin pregnancies in humans. In the Afec-Assaf sheep breed carrying the Booroola gene (hetero and homozygotes), twins account for 34% of multifetal pregnancies [8]. As in human sIUFD, the underlying mechanism leading to PLL in sheep remains unclear.

Fetus sex results from a complex prenatal interaction of genetic, hormonal, and gonadal factors. Prenatal sex-specific interactions between the dam, placenta, and fetus affect the intrauterine health of both dams and fetuses. In humans, the male fetus has been established as an independent risk factor for adverse pregnancy outcomes, including delivery distress and perinatal death [9, 10]. In human twin dichorionic pregnancies, the pregnancy outcome improves when the fetus shares the uterus with a female rather than a male co-twin. Moreover, in humans, fetus weight is reduced with male-male co-twins, which might be related to a shorter pregnancy [11], whereas in sheep, male lambs are heavier at parturition [12]. In this study, we were interested in PLL, in which the dead lambs were born within the typical birth BW range, without any visual damage or deformation. We assumed that the death of these fetuses occurred during the last trimester of pregnancy. To the best of our knowledge, no information is available on the effect of sex on fetuses’ survival in multifetal pregnancies in ewes. Therefore, we examined the association between fetal sex, PLL, dam physiological and metabolic state, and pregnancy outcome in multifetal pregnancies in ewes.

## Materials and methods

### Animals

The Volcani Center Animal Care Committee approved all procedures performed on the animals in the present study (approval no. 867/20 IL). This study included two parts, and both were conducted at the experimental flock at the Volcani Center, Rishon LeZion, Israel, which is comprised of the Afec-Assaf breed. The prolific Afec-Assaf strain is considered a dual-purpose breed and was developed by introgressing the *B* allele of the *FecB* (Booroola) locus into the Assaf breed [13]. Flock reproductive management included three 40-day breeding periods per year when each ewe was hand-mated to a single ram following hormonally synchronized estrus. In the experimental flock at the Volcani Center, lamb birth weights, sex, and vitality are determined and recorded routinely within a few hours after lambing.

### Study design–part 1

In the first part of this study, we collected the recorded lambing data, including lamb birth weights, sex, and survival for 675 lambings (litter size range 2–6; a total of 1851 lambs). Out of the 675 lambings, 325 (48.1%) were twin lambings, 222 (32.9%) were triplets lambings, 103 (15.3%) with four lambs, 22 (3.3%) with five fetuses, and three (0.4%) with six lambs. Fetus loss was defined by visible dead fetuses recorded at lambing. Data were categorized by the male-to-female ratio (**MR**): low male ratio (**LMR**)—MR < 0.5 and high male ratio (**HMR**)—MR > 0.5. A retrospective analysis was performed using the MR categories, in total: 212 LMR vs. 218 HMR lambings. Out of 675, 245 lambings were with MR = 0.5 (one female and one male, or two females and two males), and they were excluded from the analysis.

### Study design–part 2

In the second part of the study, we followed 38 multiparous Afec-Assaf ewes divided into two clusters, from 80 days in pregnancy (**DIP**) until lambing in the first cluster, and from 120 DIP until lambing in the second cluster. The ewes in this study were hand-mated following estrus synchronization. The two clusters were comprised of different animals, and in the first cluster (n = 25), ewes were mated from mid-August through mid-September and lambed from late January through mid-February, i.e., the winter season in Israel. In the second cluster (n = 13), ewes were mated in April and lambed from late August through mid-September, i.e., the summer season in Israel. The flock was kept in open sheds under non-dairy management. All ewes were housed in one pen and group-fed the same diet *ad libitum*. In this part of the study, pregnancy was determined in mated ewes on day 35 post mating by transabdominal ultrasonography (3100v, Shenzhen WellD Medical Electronics, Guangdong, China). We followed the physiological and metabolic status of the dams during the last trimester of pregnancy and the fetuses’ HR. To this end, we determined the litter size of pregnant sheep at ~60 DIP (average litter size of 2.9, range 1–6). At ~80 DIP, the selected ewes of each cluster were weighed, and their body condition score (**BCS**) was determined on a scale of 1–5 [14].

We performed ultrasound scanning (12 ewes in each cluster, a total of 24) to monitor the maternal HR (beats per minute; **bpm**) and fetal HR to follow litter vitality using Doppler ultrasound (Mindray Z6; Shenzhen Mindray Bio-Medical Electronics, Nansham, China), with a 2- to 6-MHz-range convex transducer (3C5P type; Shenzhen Mindray Bio-Medical Electronics). Scanning of the fetuses began at 80 DIP and 110 DIP for dams in the first cluster and repeated every 10 days until 138 DIP and daily from 138 DIP until lambing. In the second cluster, the scanning started on 120 DIP for both fetuses and dams, and was repeated every 10 days until 138 DIP, and then daily until lambing.

Blood samples were taken from the dams at 0630 h on the day of ultrasound scanning and from the dams and lambs at lambing. The samples were taken from the jugular vein into vacuum tubes containing lithium heparin (BD Vacutainer, Plymouth, UK) and immediately placed on ice. The plasma was separated by centrifugation for 15 min at 1000*g*, divided into two tubes, and stored at -3°C, pending analysis.

At lambing, the number of lambs born, those born alive, and the gender of the lambs were recorded. The lambs’ birth weights and crown-to-rump length (CRL) were recorded within a few hours after lambing. Dams’ body weight and BCS were recorded within 24 h after lambing. The gestation length for each ewe was calculated based on mating and lambing records.

### Chemical analyses

Plasma glucose concentration was determined using a Cobas c111 autoanalyzer (Roche Holding GmbH, Grenzach-Wyhlen, Germany) with the reagent set GLUC2, which uses two enzymatic reactions with hexokinase and glucose-6-phosphate dehydrogenase to generate a UV emission that is correlated with the sample glucose concentration; the results were calibrated against known glucose concentrations.

Plasma β-hydroxybutyrate (BHB) concentration was determined with a RANBUT D-3-Hydroxybutyrate Kit (Randox, CruMLin, UK), in which a reaction of 3-hydroxybutyrate and dehydrogenase generates a UV emission correlated with the sample BHB concentration. The samples were examined at 340 nm with an optical density reader (Spectro V-11D, MRC, Holon, Israel); the results were calibrated against known BHB concentrations. The intra- and interassay coefficients of variation for the BHB assay were 1.3 and 1.8%, respectively.

Plasma nonesterified fatty acid (NEFA) concentration was determined with a NEFA C Test Kit (Wako Chemicals GmbH, Neuss, Germany). The intra- and interassay coefficients of variation for the NEFA assay were 5.9 and 6.2%, respectively.

Plasma lactate concentration was examined in the Cobas c111 autoanalyzer with the reagent set LACT2 (Roche Holding GmbH), which uses two enzymatic reactions with lactate oxidase and peroxidase to generate a UV emission that is correlated with the sample L-lactate concentration; the results were calibrated against known L-lactate concentrations.

Plasma triglyceride (TG) concentration was determined in the Cobas c111 autoanalyzer with the reagent set TRIGL (Roche Holding GmbH), which uses four enzymatic reactions with lipase, glycerol kinase, glycerol phosphate oxidase, and peroxidase to generate a UV emission that is correlated with the sample TG concentration; the results were calibrated against known TG concentrations.

Plasma iron concentration was determined in the Cobas c111 autoanalyzer with the reagent set IRON2 (Roche Holding GmbH), which uses three reactions with acid and reductions to generate a UV emission that is correlated with the sample iron concentration; the results were calibrated against known iron concentrations.

Plasma insulin was determined by the Insulin IRMA Test Kit (Beckman Coulter, Fullerton, CA). The intra- and interassay coefficients of variation for the insulin assay were 6.7 and 6.2%, respectively.

Plasma lactate dehydrogenase (LDH) catalytic activity was examined in the Cobas c111 autoanalyzer with the reagent set LDHI2 (Roche Holding GmbH), which uses LDH to convert L-lactate to pyruvate; NAD is reduced to NADH in the process. The initial rate of NADH formation is directly proportional to the catalytic LDH activity; a UV emission was generated that was correlated with the sample LDH catalytic activity; the results were calibrated against a known LDH activity standard.

Plasma aspartate aminotransferase (AST) catalytic activity was examined in the Cobas c111 autoanalyzer with the reagent set ASTL (Roche Holding GmbH), which uses AST to convert L-aspartate and 2-oxoglutarate to oxaloacetate and L-glutamate. The oxaloacetate is then reacted with NADH in the presence of malate dehydrogenase to form NAD^+^. The rate of NADH oxidation is directly proportional to the catalytic AST activity; it generates a UV emission that is correlated with the sample AST catalytic activity; the results were calibrated against a known AST activity standard.

Plasma alkaline phosphatase (ALP) catalytic activity was examined in the Cobas c111 autoanalyzer with the reagent set ALP2S (Roche Holding GmbH), which uses ALP to convert p-nitrophenyl phosphate and water to phosphate and p-nitrophenol. The released p-nitrophenol is directly proportional to the catalytic ALP activity; it generates a UV emission that is correlated with the sample ALP catalytic activity; the results were calibrated against a known ALP activity standard.

### Statistical analysis

Part 1 –We analyzed retrospectively 675 lambings that experienced PLL; out of them, 245 lambings were with MR = 0.5 (one female and one male, or two females and two males), and were excluded from the analysis, 212 were with LMR and 218 with HMR lambings (in total = 430 were included in the statistical analysis). In addition, lambings with total litter loss were not included in test. The analysis was done as repeated measurements using the PROC MIXED procedure, version 9.2 (SAS, 2002). The odds ratio (**OR**) and PLL incidence were determined using the nominal logistic regression procedure of JMP Pro 16 (SAS, 2021).

Part two–All data were analyzed only by MR (LMR and HMR as described above). Out of 38 pregnancies, 10 were characterized as LMR and 15 as HMR. Thirteen lambings were categorized with MR = 0.5 and were excluded from the analysis. The lambing data were analyzed with the PROC GLM procedure, version 9.2 (SAS, 2002). Means were estimated using the LSMEANS statement of SAS and tested for significance by the Tukey–Kramer procedure.

The HR of fetuses and dams and metabolites in dams were analyzed by MR (LMR and HMR) as repeated measurements using the PROC MIXED procedure, version 9.2 (SAS, 2002). The following model was used: Y_ijkl_ = μ + T_i_ + E(T)_ij_ +DIP_jk_ + *E*_ijkl_, where μ = the overall mean; T_i_ = fixed effect of groups (LMR and HMR); E(T)_ij_ = ewe _j_ nested in treatment; DIP_ijk_ = day in pregnancy as a continuous variable; *E*_ijkl_ = random residual.

The effects of the cluster, litter size, and litter weight were found to be non-significant (*P* > 0.05) for all variables tested (HR and metabolites) and therefore were excluded from the model. The HR was analyzed using a similar model.

Main effects were declared significant at *P* ≤ 0.05, and tendencies were noted at 0.05 < *P* ≤ 0.10.

## Results

### Partial litter loss

A retrospective analysis of ewes with multifetal pregnancies was performed on 675 lambings (1851 lambs; litter size range 2–6), of which 191 were twin lambings. In this study, we did not analyze lambings with an equal number of male and female fetuses (MR = 0.5; n = 245). Of the 675 multifetal lambings, 212 were defined as LMR, and 218 were defined as HMR lambings. Of the 675 pregnancies, 191 lambings (28%) experienced PLL. PLL prevalence for the MR categories was higher for HMR (39.0%) compared to LMR (25.9%; *P* = 0.004; Table 1). The OR for PLL in HMR compared to LMR litters was 1.82 (95% CI 1.2–2.7; *P* < 0.005; Table 1). The survival rate was 90.0% for LMR, compared to 85.0% for HMR (*P* < 0.01; Table 1). MR did not influence pregnancy duration, total litter, and male birth weight; however, the female birth weight was 12.0% lower in the HMR than in the LMR lambings (*P* = 0.005). In addition, there was a specific gender effect on survival rate, in which the male survival rate was not affected, but the female survival rate was 9.6 percent lower in the HMR than in the LMR lambing (*P* = 0.0009; Table 1).

**Table 1:** Retrospective analysis of 430 Lambings by male ratio category.

	Male ratio*			
Parameter	LMR	HMR	SEM	*P-*value
n	212	218		
Average Litter size	2.9	2.8	0.09	0.42
Pregnancy duration (days)	144.8	145.1	0.22	0.42
Litter survival rate	0.90	0.85	0.01	0.008
PLL incidence (%)	25.9	39.0	–	0.004
OR for PLL^†^	–	1.82	–	0.004
Total litter weight (kg)	10.9	11.3	0.25	0.35
Male lamb BW (kg)	4.1	4.2	0.11	0.55
Female lamb BW (kg)	4.0	3.5	0.09	0.005
Male survival rate	0.86	0.85	0.02	0.58
Female survival rate	0.90	0.82	0.02	0.0009

^*^LMR–low male ratio (MR < 0.5); HMR–high male ratio (MR > 0.5).

^†^Odds ratio for partial litter loss of MR >0.5 compared to MR <0.5 twins (CI 95%).

PLL, partial litter loss; BW, body weight

### Second part – controlled study

In this part, we followed the physiology and metabolic state of 38 multifetal dams and their fetuses from 80 DIP until lambing. We analyzed the data according to the litter MR as defined above (n = 25) and found that MR did not influence pregnancy duration, dams’ BW, or BCS at 80 DIP or lambing (Table 2). The litter survival rate was decreased by 32 percentage units for HMR compared to LMR litters (*P* = 0.02). MR did not affect total litter weight, but female lambs’ BW was 1.3-fold higher (*P* = 0.03), and CRL tended to be longer in 7.6% (*P* = 0.06) in LMR vs. HMR litters. Male lambs’ BW was 19% higher (*P* = 0.13) for LMR than for HMR litters. Male lambs’ CRL was not affected by MR (Table 2).

**Table 2:** Mean of ewe and lamb pregnancy outcomes by male ratio category.

	Male ratio*			
Parameter	LMR	HMR	SEM	*P-*value
n	10	15		
Pregnancy duration (days)	145.1	144.9	0.7	0.80
Dam BW at 80 DIP (kg)	96.4	88.8	2.9	0.08
Dam BCS at 80 DIP	3.4	3.1	0.1	0.13
Dam BW postpartum (kg)	92.7	83.7	2.6	0.03
Dam BCS postpartum	2.8	2.5	0.2	0.15
Litter survival rate	0.94	0.62	0.09	0.02
Total litter weight (kg)	11.6	10.6	0.71	0.33
Male lamb BW (kg)	4.4	3.7	0.29	0.13
Female lamb BW (kg)	4.5	3.6	0.25	0.03
Male lamb CRL (cm)	51.9	50.5	0.01	0.49
Female lamb CRL (cm)	53.8	50.0	0.01	0.06

^*^LMR–low male ratio (MR < 0.5); HMR–high male ratio (MR > 0.5).

DIP, days in pregnancy; BW, body weight; CRL, crown-to-rump length.

### Dam and fetus physiology, and plasma metabolite, insulin, and enzyme concentrations

**Fig 1:**
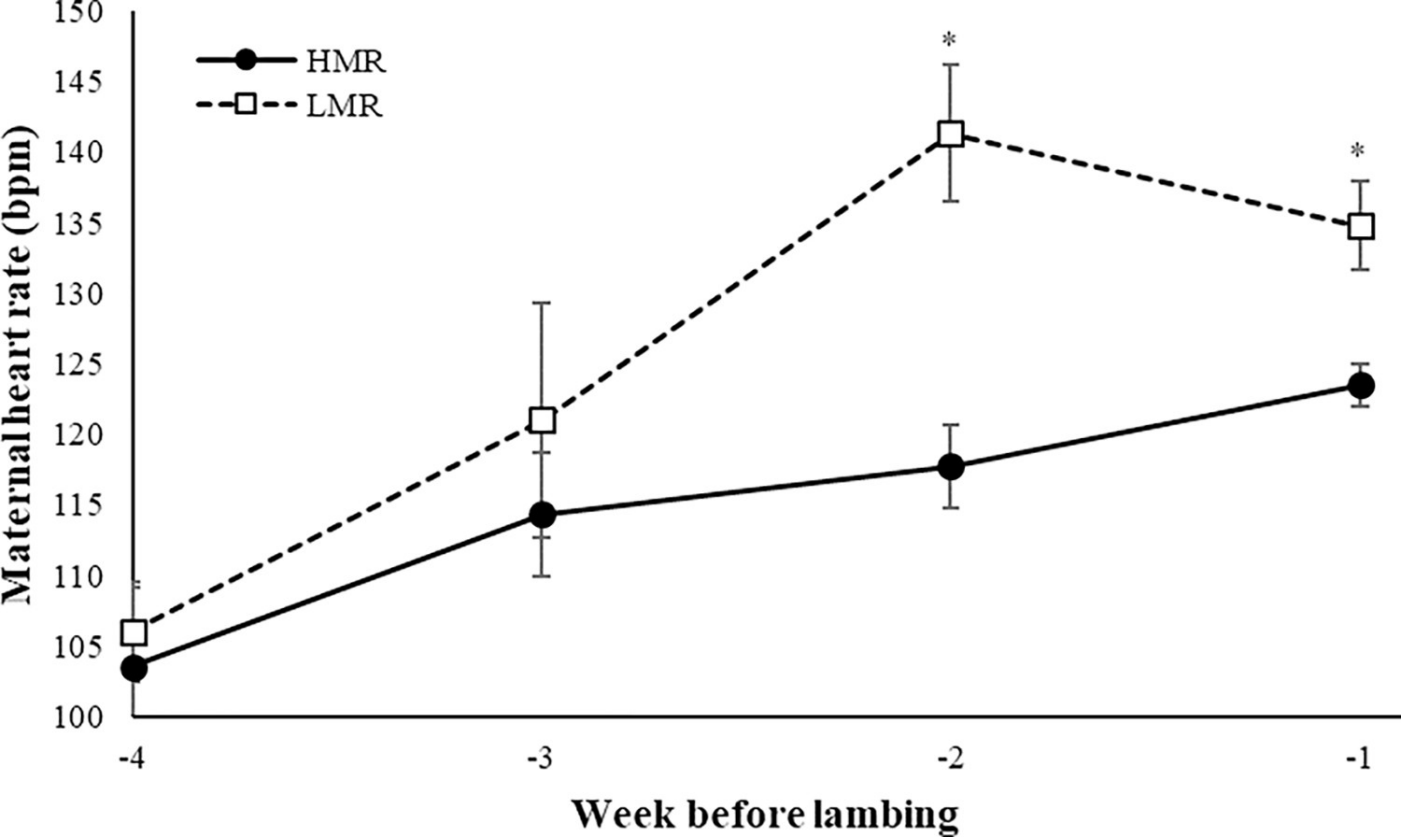
Dam’s heart rate during the last 4 weeks of pregnancy by male ratio category. Ewe carrying a litter with a male-to-female ratio >50% (HMR; black circle) or <50% (LMR; white square). Asterisks above the bars indicate significant effect of treatments as follows: **P < 0.01, *P < 0.05.

The mean maternal HR in LMR pregnancies was higher by 9.4% than in the HMR pregnancies at DIP ≥ 110 (133.3 vs. 121.8 bpm, respectively; *P* = 0.03; Fig 1). However, MR did not affect the fetus’s mean HR in the last trimester (148.4 vs. 150.6 bpm, respectively; *P* = 0.58).

**Fig 2:**
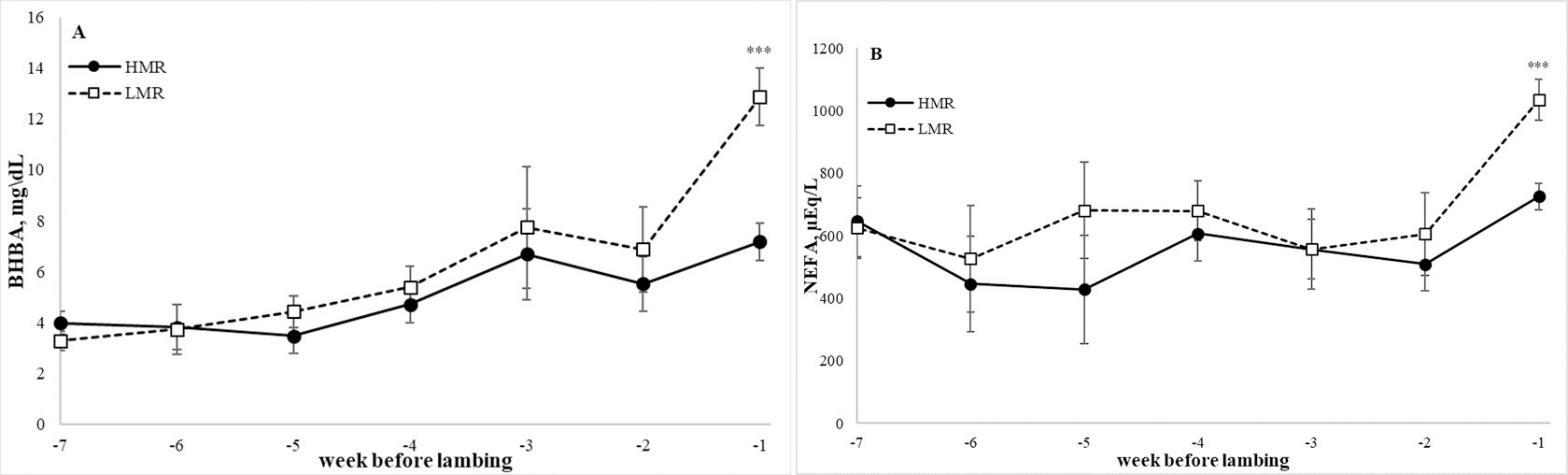
Plasma concentration of β-hydroxybutyrate (BHB;A) and nonesterified fatty acid (NEFA; B) in the last 7 weeks of pregnancy in prolific ewes by male ratio category. Ewe carrying a litter with a male-to-female ratio >50% (HMR; black circle) or <50% (LMR; white square). Asterisks above the bars indicate significant effect of treatments as follows: ***P < 0.001, **P < 0.01, *P < 0.05.

In the last trimester, plasma glucose concentration in ewes was unaffected by MR (*P* = 0.14), with no significant difference in insulin concentration. Plasma BHB and NEFA concentrations were 31% (*P* < 0.009; Table 3 and Fig 2A) and 20% (*P* = 0.03; Table 3 and Fig 2B) lower in the HMR vs. LMR ewes, respectively. A greater difference between HMR and LMR groups in plasma glucose concentrations was observed in the last week of pregnancy (51.4 vs. 43.8, respectively; *P* = 0.007) and BHB (7.2 vs. 12.8, respectively; *P* < 0.0001; Fig 2A). There were no significant differences between HMR and LMR dams in plasma concentrations of lactate, TG, iron, or total AST, LDH, and ALP activity.

**Table 3:** Dams and fetuses HR, and ewe plasma metabolites, insulin, and enzymes during the last trimester, not including day of lambing, by male ratio.

	Male ratio*			
Parameter	LMR	HMR	SEM	*P-*value
Glucose (mg/dL)	48.0	51.5	1.6	0.15
BHB (mg/dL)	8.7	6.0	0.65	0.009
NEFA (μEq/L)	830.1	663.4	40.5	0.03
Insulin (μIU/mL)	58.4	44.7	7.3	0.20
Lactate (mmol/L)	0.71	0.66	0.04	0.45
TG (mg/dL)	24.9	24.0	2.0	0.76
AST (units/L)	65.2	65.6	2.95	0.93
LDH (units/L)	283.3	295.4	9.0	0.38
ALP (units/L)	58.3	57.1	5.0	0.86
Iron (μmol/L)	29.0	28.4	0.9	0.67

^*^LMR–low male ratio (MR < 0.5); HMR–high male ratio (MR > 0.5).

BHB, β-hydroxybutyrate; NEFA, nonesterified fatty acid; TG, triglyceride; AST, aspartate aminotransferase; LDH, lactate dehydrogenase; ALP, alkaline phosphatase.

## Discussion

We examined the effect of multifetal litters’ MR on PLL in late pregnant ewes. We observed an increased risk for PLL with increasing litter MR. We also observed reduced HR and lowered BHB and NEFA concentrations in dams carrying HMR vs. LMR litters during the last trimester.

In our retrospective study, the PLL rate in multifetal pregnancies (3–6 fetuses) was 28%, and in twin pregnancies, it was 16.8%. In human twin pregnancies, the **sIUFD** prevalence was estimated at 3.7–6.8% of pregnancies [15], and in sheep, in a continuous monitoring study, PLL from 85 DIP to birth was estimated at 17.7% [3], which is very similar to our findings in twin pregnancies. However, it should be noted that in this study, since we did not have pre-lambing information, the PLL was determined only at lambing, which means that only visible dead fetuses were recorded. We suggest that the difference between humans and sheep in PLL prevalence in twin pregnancies is due to the difference in the care and monitoring frequency of the pregnant female. Among other reasons, we assume that the optional early controlled Cesarean surgery in humans enhances newborns’ survival.

We observed a strong correlation between PLL and the litter’s MR (25.9 vs. 39.0% in LMR and HMR, respectively). In humans, male fetuses have an adverse effect on pregnancy outcomes, including hypertension [10], preterm delivery [16], gestational diabetes mellitus, macrosomia, cord prolapse, nuchal cord, umbilical cord notes, a higher rate of Cesarean sections, non-reassuring fetal HR [17, 18], low Apgar scores, delivery distress, and perinatal death [9, 10]. In a systemic review and meta-analysis of over 3 million human births, the authors suggested that male fetuses increase the relative risk for stillbirth in all populations by about 10% [19]. In addition, analysis of 423,033 singleton pregnancies revealed an increased risk for operative delivery due to fetal distress (OR = 1.36; 95% CI 1.33–1.39), low 5-min Apgar score (0–3; OR = 1.38; 95% CI 1.32–1.44), and perinatal death (OR = 1.38; 95% CI 1.32–1.44) in male compared to female fetuses [9]. It was also reported in human twin pregnancies, that the outcome improves when the fetus shares the womb with a female rather than a male co-twin [11]. In sheep twin pregnancies, it has been reported that when there are female-male co-twins, the female’s weight at lambing is reduced, as is its lifetime breeding success [20], which was also found in our study, in which the female birth weight was 12.5% lower in HMR lambings (Table 1). IUGR and low BW at birth in multifetal pregnancies in sheep were associated with limited placental transport of nutrients from the dam to fetuses through the uteroplacental complex [6] and with lower placentome mass [4]. Placental amino acid transport is reduced in the presence of testosterone in rats [21], and in the human placenta, androgen receptor activity has been reported to be affected by the fetus’s sex [22], which may partly explain the low BW at birth of female in HMR lambings. Sex-specific changes in the maternal placenta–fetal axis activity affected by hormonal, genetic, or epigenetic mechanisms are assumed to be related to pregnancy outcome [10].

The maternal HR, but not the fetal HR, was affected by MR in the last trimester in the current study. However, data on the association between maternal HR and fetal gender are limited. For example, Gonçalves et al. [23] reported increased maternal HR during labor with males compared to female fetuses in humans. Controversial results have been reported on the influence of fetal gender on fetal HR, where some studies agree with our result, reporting no gender-specific differences in fetal HR [23, 24], and others reported higher mean fetal HR in females [25, 26].

No effect of MR was observed on dams’ glucose concentration, but BHB and NEFA concentrations were lower in HMR than in LMR, which suggests a better metabolic status for the dams carrying HMR litters. Higher fasting glucose concentration during pregnancy, but below the gestational diabetes mellitus diagnostic threshold, has been reported for singleton male pregnancies [27, 28]. Geng et al. [27] reported elevated fasting pregnancy glucose for pregnancies with males but not higher insulin concentrations, which is partly in agreement with our findings. They also reported no difference in glucose concentration during the oral glucose tolerance test [27]. However, Retnakaran et al. [28] reported elevated glucose concentration during the oral glucose tolerance test with no difference in insulin sensitivity or insulin resistance for male-bearing pregnancies. Although the underlying mechanism is unclear, this suggests a reciprocal interaction between the gravid uterus and maternal metabolism.

## Conclusions

Our results demonstrate a litter-sex effect on dams’ physiology and metabolic activity in sheep. Dams carrying an HMR litter have a better metabolic and physiological state at the end of the pregnancy, but the pregnancy outcome is impaired. The lower vitality of HMR litters might be part of the uncontrolled intrauterine growth restriction mechanism. We suggest that sheep can be a supportive animal model for studying sIUFD and gender impact on pregnancy outcome, as suggested previously [29].
